# Acute on chronic bilateral subdural hematoma presenting with acute complete flaccid paraplegia and urinary retention mimicking an acute spinal cord injury: a case report

**DOI:** 10.1186/s13104-017-2969-y

**Published:** 2017-11-28

**Authors:** H. M. M. T. B. Herath, Anne Thushara Matthias, Aruna Kulatunga

**Affiliations:** National Hospital, Colombo, Sri Lanka

**Keywords:** Bilateral chronic subdural hematoma, Acute flaccid paraplegia, Urinary retention

## Abstract

**Background:**

A subdural hematoma refers to a collection of blood between the dura and the arachnoid membranes and is classified into acute, sub acute and chronic. Subdural hematoma has been referred to as the “great neurologic imitator” as it can mimic many neurological conditions.

**Case presentation:**

Forty-three year old Sri Lankan female presented 2 weeks following traumatic head injury with bilateral flaccid complete paraplegia and urinary retention. Her non-contrast computer tomography of the brain revealed bilateral acute, chronic subdural hematomas. Both subdural hematomas were aspirated and she recovered completely.

**Conclusions:**

Chronic subdural hematoma can present in many different unusual ways including bilateral complete paraplegia and acute urinary retention mimicking acute spinal cord pathology. The exact mechanism of this clinical presentation is not clear and may be due to direct compression of the motor cortex to the falx or due to compression of the anterior cerebral artery due to subfalcine herniation. This case illustrates the importance of considering subdural hematoma as a rare cause for acute paraplegia and the importance of performing a computer tomography scan in traumatic brain injury when indicated. Failure to consider non-spinal causes of paraplegia results in potential mismanagement. According to our knowledge this is the first case report of acute on chronic subdural hematoma presenting as acute flaccid complete paraplegia with urinary retention.

## Background

A subdural hematoma (SDH) refers to a collection of blood between the dura and the arachnoid membranes. SDH is classified into three types: acute, sub acute and chronic. Acute SDH is usually caused in head injury and is symptomatic within 24 h of injury [1]. Chronic SDH generally develop after the initial meningeal trauma by complete encapsulation of the clot [2] and become symptomatic more than 2 weeks after the initial injury. SDH has been referred to as the “great neurologic imitator” [3, 4] as it can mimic stroke, dementia, parkinson’s. Here we describe a case of a young female presenting 2 weeks following traumatic head injury with bilateral flaccid complete paraplegia with urinary retention. Her non-contrast computer tomography (CT) brain revealed bilateral acute on chronic SDH. The presence of acute bilateral lower limb paraplegia with urinary retention is usually suggestive of a spinal cord lesion and cerebral causes are rare. This is a rare presentation of SDH mimicking acute spinal cord injury and illustrates the need to consider the entire neuro-axis in the differential diagnosis of acute paraplegia.

## Case presentation

Forty-three year old previously well Sri Lankan female presented with acute onset bilateral lower limb complete weakness and acute urinary retention of 2 days duration. She did not complain of numbness of lower limbs or weakness of upper limbs. There was no backache. Sixteen days back she had met with a road traffic accident and was struck by a bus which accounted for high-energy head injury. She has fallen on the ground and lost consciousness for 15 min and experienced retrograde amnesia. There was no injury to the spine. Subsequently in the hospital she had persistent headache with several episodes of vomiting. On discharge she did not have any neurological deficit or signs of basal skull fracture (haemotympanum, ‘panda’ eyes, cerebrospinal fluid leakage from the ear or nose, bruising behind the ear). She did not have any significant past medical or surgical history or a bleeding diathesis. She was not taking any narcotic analgesics.

On examination she was not pale. Cardiovascular, respiratory and abdominal examination was normal with a heart rate of 80 beats/min and a blood pressure of 130/80 mmHg. She was conscious with a Glasgow coma scale of 15/15 and cranial nerve examination was normal without ophthalmoplegia or nystagmus. Fundoscopy did not show papilledema. Tone, power, reflexes and sensation of the upper limbs were normal. There were no cerebellar signs. Lower limbs were flaccid with power grade 0. Knee and ankle reflexes were diminished and plantar response was flexor. All sensory modalities of the lower limb were preserved. She was catheterized for acute urinary retention before transferring from the local hospital so did not have palpable bladder. Anal tone was normal and superficial abdominal reflexes were present. There were no tender areas over the spine.

**Fig. 1:**
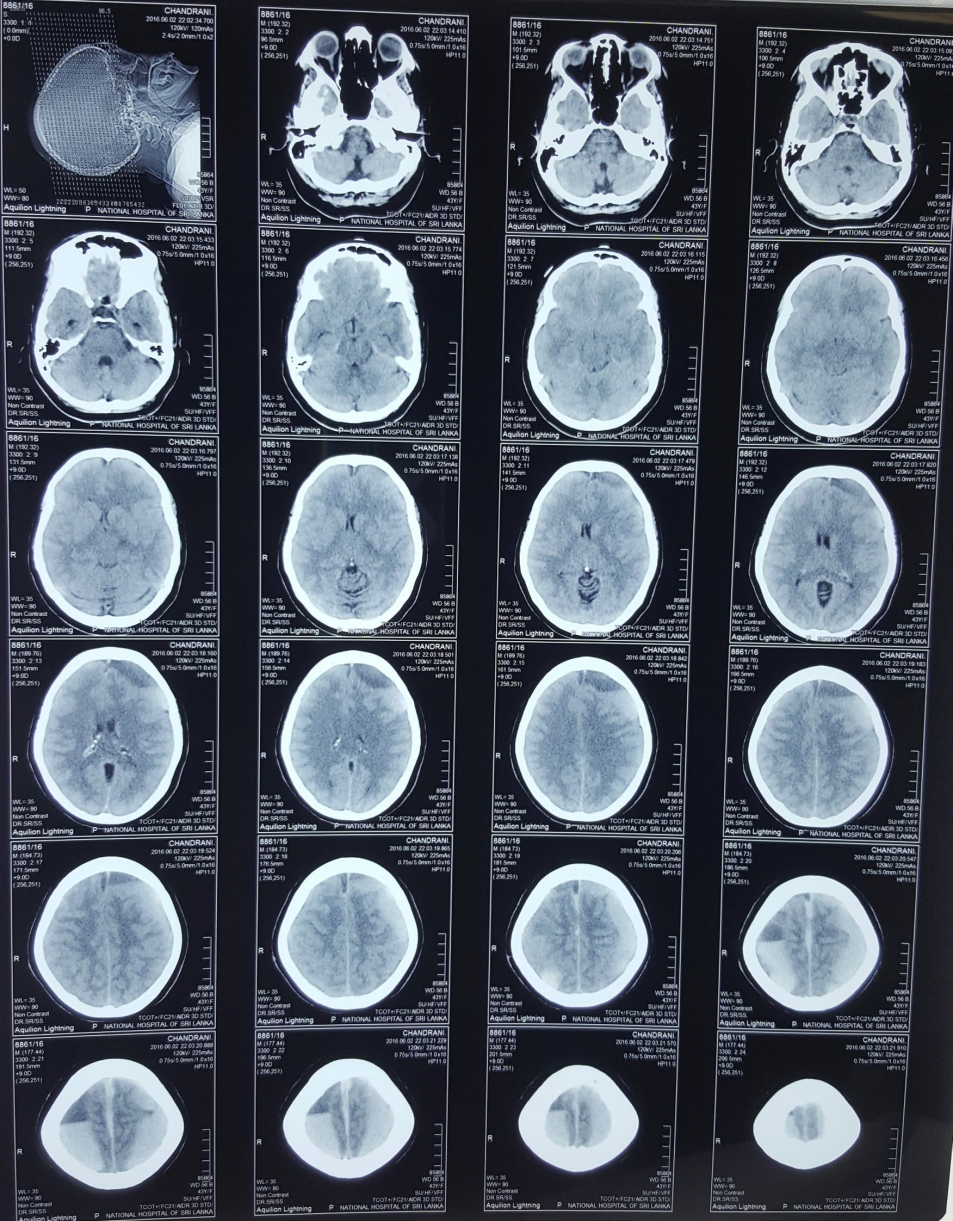
Non-contrast computer tomography of the brain showing bilateral acute on chronic sub dural hematoma. Left sided sub dural hematoma was over the frontal, parietal, occipital lobes and right-sided sub dural hematoma was located over the parietal and occipital lobes. The sub dural hematoma extended into the interhemispheric space at the vertex but was not seen in the deeper part of the brain. The left sided sub dural hematoma was larger and there was a mild midline shift and subfalcine herniation to the right side

Initial whole blood count, liver function tests, renal function tests, clotting profile and basic investigations were normal. X-ray of the thoracolumbar spine did not reveal any abnormality. Non-contrast CT brain showed bilateral acute on chronic SDH. Left sided SDH was over the frontal, parietal, occipital lobes and right-sided SDH was located over the parietal and occipital lobes (Fig 1). The SDH extended into the interhemispheric space at the vertex but was not seen in the deeper part of the brain. There were no intracranial hemorrhages. Because the left sided SDH was larger there was a mild midline shift and subfalcine herniation to the right side.

**Fig. 2:**
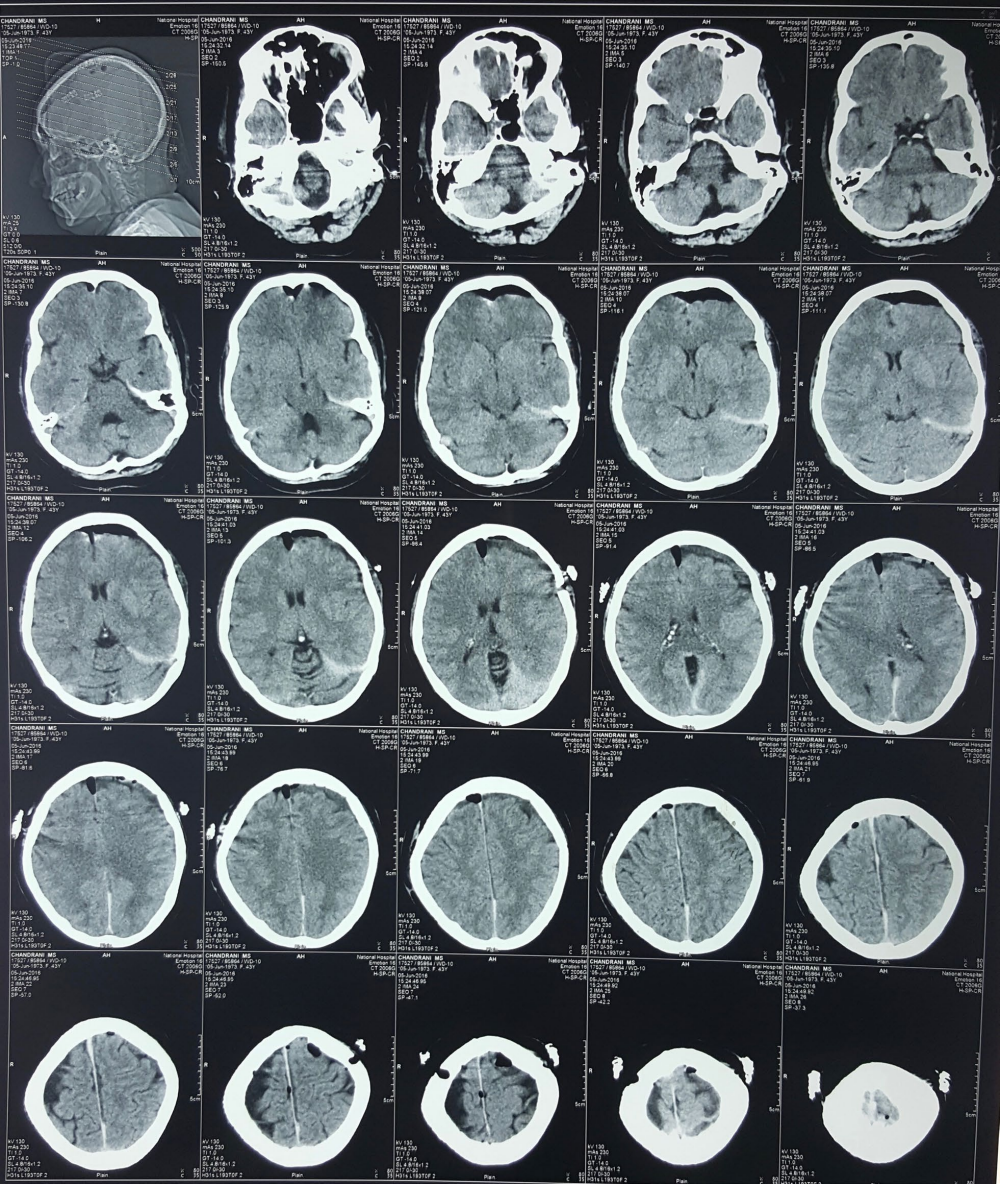
Post operative non-contrast computer tomography of the brain following bilateral burr hole aspiration

Urgent neurosurgical referral was made and she underwent bilateral burr hole aspiration of 60 ml and 40 ml of blood from left to right, respectively. Within 12 h she was able to move the left lower limb and within 24 h her right lower limb as well. She completely regained her lower limb power within 48 h. Postoperative non-contrast CT brain was repeated (Fig. 2). Headache resolved after 1 week and urinary catheter removed successfully after 1 week.

## Discussion and conclusion

The causes of acute SDH are variable. Head trauma is the most common cause of acute SDH with the majority of cases related to motor vehicle accidents, falls, and assaults. Here tearing of the bridging veins, that drain from the surface of the brain to the dural sinuses, from high-energy, short-duration force causes bleeding into the space between the arachnoid membranes and dura [5]. Twenty to thirty percent of SDH are due to arterial rupture [6]. Low cerebrospinal fluid pressure can also cause SDH [7]. Other predisposing conditions for the development of SDHs are cerebral atrophy (in elderly, chronic alcohol abusers [8], and those with previous traumatic brain injury [9]), anticoagulation [10], coagulopathy [11]. Uncommon etiologies are aneurysmal rupture [12], arteriovenous malformation [13], meningioma [14], dural metastases [15], neurosurgical procedures, cocaine abuse [16], sarcoidosis [17] and local infection [18].

Pathophysiology of chronic SDHs is complex and there is slow evolution and development of the hematoma over a long period of time [18] A thick outer membrane is formed around the heamatoma resulting in complete encapsulation of the it approximately over 2 weeks [2].

The initial presentation of SDH has a spectrum of clinical manifestations and so has been referred to as the “great neurologic imitator” [3, 4] Acute SDH presents with a clear history of trauma followed by headache and altered level of consciousness or coma in approximately 50% of cases. In approximately 12–38% of patients there is a transient “lucid interval” followed by a progressive neurologic decline to coma [5]. Chronic subdural hematoma present as insidious onset of headaches, nausea and vomiting, light-headedness, mental status changes like cognitive impairment, apathy, somnolence, dementia, mild confusion, and various levels of decreased consciousness [4, 19]. Global neurological deficits are more common than focal deficits [20]. Focal deficits may be either ipsilateral (due to lateral displacement of the midbrain caused by increased intracranial pressure resulting in compression of the contralateral cerebral peduncle against the free edge of the tentorium [5]) or contralateral to the side of the SDH. Contralateral (as a result of direct compression of cortex underlying the hematoma) SDH also can mimic stroke or transient ischemic attacks with symptoms including aphasia, hemiparesis, and hemisensory defects and possible theories to explain these include decreased regional blood flow from intermittent vessel compression, vascular displacement from parenchymal swelling, and electrophysiologic aberrations such as seizures or cortical depression [21–23].

Rare presentations of SDH include Parkinson’s disease [24, 25], seizures [4, 26, 27] Normal pressure hydrocephalus, isolated oculomotor nerve palsy [28] cranial nerve palsies [4] and cerebellar signs [29]. Only few case reports on SDH presenting as quadriparesis [30] and paraparesis has been described in the literature. One report describes a 69-year-old male with intermittent, proximal, painless paraparesis and normal sensibility who had bitemporally located chronic subdural haematoma. His neurologic deficits regressed totally within 12 h postoperatively [31] Another 58-year-old male patient who has presented with weakness of lower limbs and retention of urine and constipation and found to have a spontaneous chronic subdural haematoma [32] Shields et al. report a patient with bilateral isodense subdural hematomas who presented with paraparesis [33]. In one case report 72 years old man presented with progressive spastic paraplegia as an unusual presentation of bilateral chronic subdural hematoma [34]. In our case report this patient presented with acute onset bilateral flaccid complete paraplegia with a power of zero and urinary retention mimicking acute spinal injury with spinal shock. This happened 15 days later following traumatic brain injury and CT revealed bilateral acute on chronic SDH with interhemispheric bleed and she improved rapidly with the evacuation of SDH.

The presence of a bilateral lower limb paraplegia and urinary retention directs us towards spinal cord pathology; however, the proximity of the two lower limb motor homunculus in the brain beside falx cerebre provides cerebral causes with the potential for bilateral symptoms. Several theories have been suggested to explain this clinical presentation. In the case described by Schaller et al. the chronic SDH possibly lead to impairment of cerebral blood flow in the area of the middle cerebral artery causing intermittent, painless paraparesis. Small changes in systemic blood pressure lead to changes in cerebral perfusion pressure due to vessel compression by the haematoma, thus explaining the intermittent character of the clinical presentation [31]. Lesoin et al. present a case of bilateral SDH with quadriparesis with the motor deficit in the arms is explained by direct compression or distortion of the cerebral hemispheres; however, motor weakness in the legs cannot be explained because the cortical areas responsible for the lower limbs would be relatively protected from the direct effect of compression. The explanation given for paraparesis is ischemia of the appropriate cortical areas supplied by the anterior cerebral artery [30].

The other possibility is the subdural heamatoma leading to subfalcine herniation, as seen in our patient, which can cause injury to neural tracts directly by compressing the contralesional white matter or by compressing branches of the cerebral artery and secondarily inducing ischemic infarcts [35]. The anterior cerebral artery forms at the termination of the internal carotid artery and arches anteromedially to pass anterior to the genu of the corpus callosum and lies in the cingulate sulcus. It supplies the whole of the medial surfaces of the frontal lobe which has the motor homunculus of the lower limb. Anterior cerebral artery can get compressed by subfalcine herniation and cause reduce blood supply to this area leading to lower limb weakness. One case report describes a patient with left hemiparesis and right interhemispheric subdural heamatoma and angiography demonstrated a lateral displacement of the right callosomarginal artery and an avascular area between the falx and the callosomarginal artery [36]. Paraparesis has been seen in ruptured anterior cerebral artery territory aneurysms [37]. Other possibility is direct compression of the motor strip of the lower limbs to the falx due to increased intracranial pressure leading to bilateral leg weakness as seen in parasagittal meningioma. Koyama et al. [38] and Vaz et al. [39] describe traumatic bilateral interhemispheric subdural hematomas causing paraparesis. Spastic hemiplegia has been associated with haemorrhagic lesions in the parasagittal white matter in traumatic brain injury [40] and these suggests that parasagittal pathology can lead to paraparesis.

The micturition control center is located in the frontal lobe of the brain and damage to frontal lobe usually result in loss of voluntary control of the normal micturition reflex. But frontal lobe pathology can lead to urinary retention as well [41, 42]. This might be the cause for the urinary retention in our patient. The patient was not given narcotics for analgesia which can also cause urinary retention. The exact mechanism of chronic SDH causing bilateral lower limb weakness and urinary retention is not completely understood and represent a gray zone for further exploration.

The other important fact is the importance of CT brain in managing head injury. According to NICE guidelines in performing a CT head scan in head injury she should have undergone CT scan because she had more than one episode of vomiting, loss of consciousness with dangerous mechanism of injury and more than 30 min retrograde amnesia to events immediately before the head injury. Finally this case illustrate the importance of considering SDH as a rare cause for acute paraplegia with urinary retention and failure to consider non-spinal causes of paraplegia results in potential mismanagement.

## Abbreviations

SDH: sub dural hematoma
CT: computer tomography
NICE: National Institute for Health and Care Excellence

## References

[1] Miller JD, Nader R (2014). Acute subdural hematoma from bridging vein rupture: a potential mechanism for growth. J Neurosurg.

[2] Mayer S, Rowland L, Rowland L (2000). Head injury. Merritt’s neurology.

[3] Potter JF, Fruin AH (1977). Chronic subdural hematoma—the “great imitator”. Geriatrics.

[4] Luxon LM, Harrison MJ (1979). Chronic subdural hematoma. Q J Med.

[5] Victor M, Ropper A, Victor M, Ropper A (2001). Craniocerebral trauma. Adams and Victor’s principles of neurology.

[6] Gennarelli TA, Thibault LE (1982). Biomechanics of acute subdural hematoma. J Trauma.

[7] Ezri T (2002). Intracranial subdural hematoma following dural puncture in a parturient with HELLP syndrome. Can J Anaesth.

[8] Sonne NM, Tonnesen H (1992). The influence of alcoholism on outcome after evacuation of subdural haematoma. Br J Neurosurg.

[9] Doherty DL (1988). Posttraumatic cerebral atrophy as a risk factor for delayed acute subdural hemorrhage. Arch Phys Med Rehabilit.

[10] Mattle H (1989). Anticoagulation-related intracranial extracerebral haemorrhage. J Neurol Neurosurg Psychiatry.

[11] Seckin H (2006). Chronic subdural hematoma in patients with idiopathic thrombocytopenic purpura: a case report and review of the literature. Surg Neurol.

[12] Barton E, Tudor J (1982). Subdural haematoma in association with intracranial aneurysm. Neuroradiology.

[13] Rengachary SS, Szymanski DC (1981). Subdural hematomas of arterial origin. Neurosurgery.

[14] Okuno S (1999). Falx meningioma presenting as acute subdural hematoma: case report. Surg Neurol.

[15] Laigle-Donadey F (2005). Dural metastases. J Neurooncol.

[16] Alves OL, Gomes O (2000). Cocaine-related acute subdural hematoma: an emergent cause of cerebrovascular accident. Acta Neurochir.

[17] de Tribolet N, Zander E (1978). Intracranial sarcoidosis presenting angiographically as a sub-dural hematoma. Surg Neurol.

[18] Traynelis VC (1991). Chronic subdural hematoma in the elderly. Clin Geriatr Med.

[19] Fogelholm R, Heiskanen O, Waltimo O (1975). Chronic subdural hematoma in adults. Influence of patient’s age on symptoms, signs, and thickness of hematoma. J Neurosurg.

[20] Adhiyaman V (2002). Chronic subdural haematoma in the elderly. Postgrad Med J.

[21] Mishriki YY (1999). Subdural hematoma mimicking a transient ischemic attack due to antihypertensive medication. South Med J.

[22] Wilkinson CC, Multani J, Bailes JE (2001). Chronic subdural hematoma presenting with symptoms of transient ischemic attack (TIA): a case report. W V Med J.

[23] Moster ML, Johnston DE, Reinmuth OM (1983). Chronic subdural hematoma with transient neurological deficits: a review of 15 cases. Ann Neurol.

[24] Wiest RG, Burgunder JM, Krauss JK (1999). Chronic subdural haematomas and Parkinsonian syndromes. Acta Neurochir.

[25] Ellis GL (1990). Subdural hematoma in the elderly. Emerg Med Clin N Am.

[26] Rubin G, Rappaport ZH (1993). Epilepsy in chronic subdural haematoma. Acta Neurochir.

[27] Cameron MM (1978). Chronic subdural haematoma: a review of 114 cases. J Neurol Neurosurg Psychiatry.

[28] Phookan G, Cameron M (1994). Bilateral chronic subdural haematoma: an unusual presentation with isolated oculomotor nerve palsy. J Neurol Neurosurg Psychiatry.

[29] Stendel R (2002). Spontaneous bilateral chronic subdural haematoma of the posterior fossa. Case report and review of the literature. Acta Neurochir.

[30] Lesoin F (1983). Quadriparesis as an unusual manifestation of chronic subdural haematoma. J Neurol Neurosurg Psychiatry.

[31] Schaller B (1999). Intermittent paraparesis as manifestation of a bilateral chronic subdural hematoma. Schweiz Med Wochenschr.

[32] Sangondimath G (2015). A rare case of chronic subdural haematoma presenting with paraparesis: a case report and review of literature. J Clin Orthop Trauma.

[33] Shields CB, Stites TB, Garretson HD (1980). Isodense subdural hematoma presenting with paraparesis: case report. J Neurosurg.

[34] Kyriacou A, Lim C, Ahmed A (2012). Bilateral chronic subdural hematoma: an unusual presentation with progressive spastic paraplegia. Arch Hell Med.

[35] Byard RW (2013). Patterns of cerebral and cerebellar herniation. Forensic Sci Med Pathol.

[36] Takeda N (1988). Three cases of acute interhemispheric subdural hematoma. No Shinkei Geka.

[37] Endo H, Shimizu H, Tominaga T (2005). Paraparesis associated with ruptured anterior cerebral artery territory aneurysms. Surg Neurol.

[38] Koyama S, Nishimura T (1990). A case of bilateral interhemispheric subdural hematoma. No Shinkei Geka.

[39] Vaz R (1991). Traumatic interhemispheric subdural haematomas. Acta Neurochir.

[40] Masuzawa H (1994). Parasagittal white matter shearing injury (so-called gliding contusion): possible radiological evidence of spastic hemiplegia in diffuse axonal injury. No Shinkei Geka.

[41] Lang EW, Chesnut RM, Hennerici M (1996). Urinary retention and space-occupying lesions of the frontal cortex. Eur Neurol.

[42] Vander T, Ifergane G (2004). Transient postictal urinary retention: presentation of three cases. Eur J Neurol.

